# The Neurocognitive Effects of *Bacopa monnieri* and Cognitive Training on Markers of Brain Microstructure in Healthy Older Adults

**DOI:** 10.3389/fnagi.2021.638109

**Published:** 2021-02-22

**Authors:** Grace M. McPhee, Luke A. Downey, Keith A. Wesnes, Con Stough

**Affiliations:** ^1^Centre for Human Psychopharmacology, Swinburne University of Technology, Melbourne, VIC, Australia; ^2^Institute for Breathing and Sleep, Austin Health, Melbourne, VIC, Australia; ^3^Wesnes Cognition Ltd., Streatley, United Kingdom; ^4^University of Exeter Medical School, University of Exeter, Exeter, United Kingdom

**Keywords:** *Bacopa monnieri*, Brahmi, cognitive training, cognitive aging, brain microstructure, diffusion weighted imaging

## Abstract

*Bacopa monnieri* (BM) is a herbal supplement that increases signaling molecules implicated in synaptogenesis. Combined with cognitive stimulation, it may be a viable supplement to enhance long-term potentiation (LTP) and improve cognitive health in older adults. This randomized, double-blind, placebo-controlled trial asked 28 healthy adults aged over 55 years to complete cognitive training (CT) 3 hours weekly for 12 weeks. Fifteen consumed a standardized extract of BM and 13 consumed a placebo daily. Cognitive tasks, life-satisfaction, memory complaints and mood were assessed, and bloods analyzed for serum brain-derived neurotrophic factor (BDNF) before and after 12-weeks of the intervention. Diffusion tensor imaging (DTI) and neurite orientation dispersion and density imaging (NODDI) in gray (GM) and white matter (WM) were also analyzed. Results demonstrated slower reaction time in an image discrimination task in the BM group and faster reaction time in a spatial working memory task (SWM-O RT) in the placebo group. Mean accuracy was higher in the BM group for these tasks, suggesting a change in the speed accuracy trade-off. Exploratory neuroimaging analysis showed increased WM mean diffusivity (MD) and GM dispersion of neurites (orientation dispersion index, ODI) and decreased WM fractional anisotropy (FA) and GM neurite density (ND) in the BM group. No other outcomes reached statistical significance. An increase in ODI with a decrease in MD and ND in the BM group may indicate an increase in network complexity (through higher dendritic branching) accompanied by dendritic pruning to enhance network efficiency. These neuroimaging outcomes conflict with the behavioral results, which showed poorer reaction time in the BM group. Given the exploratory outcomes and inconsistent findings between the behavioral and neuroimaging data, a larger study is needed to confirm the synaptogenic mechanisms of BM.

## Introduction

Aging is associated with poorer cognitive outcomes (Singer et al., 2003; Deary and Der, 2005; Schaie and Willis, 2010; Singh-Manoux et al., 2012) and wide-scale re-organization of neuronal networks that decrease the efficiency of neuronal interconnectivity (Hedden and Gabrieli, 2004; Grady, 2012). This may be due to the reduced capacity for activity-dependent long-term potentiation (LTP) and a decrease in synapse number (Burke and Barnes, 2006).

Cognitive training (CT) may enhance LTP by encouraging persistent neuronal activity (Rebok et al., 2007). As neuronal activity causes the excitation of presynaptic neurons, which in turn, strengthens the connection between neurons (termed synaptogenesis), this may lead to enhanced cognitive ability after training (Zito and Svoboda, 2002; Nicholson and Geinisman, 2006). CT has shown to improve (Chapman et al., 2015; Lampit et al., 2015) and/or maintain (Cao W. et al., 2016; Luo et al., 2016) brain function/connectivity in older adults. Differences in brain structure have been observed between those who complete CT compared to controls, (Lövdén et al., 2010; Engvig et al., 2012; Chapman et al., 2015; Cao X. et al., 2016), and this difference is due to the maintenance of white matter (WM) microstructure after CT compared to deterioration in these regions in controls (McPhee et al., 2019). CT may, therefore, provide a means for protection from age-related brain structural and functional deterioration, but additional interventions are needed to produce greater benefits.

*Bacopa monnieri* (BM) is a herbal extract of the water hyssop plant. Animal studies have demonstrated BM increases signaling molecules involved in synapse formation and maintenance (Aguiar and Borowski, 2013), including increases in protein-kinase activity (Saraf et al., 2010; Prisila et al., 2012), neurotrophins (Preethi et al., 2016; Kwon et al., 2018), and phosphorylated CREB (Preethi et al., 2012; Kwon et al., 2018). These molecules are integral to synaptic plasticity, including the formation of new dendritic spines (Jourdain et al., 2003), increasing the concentration of post-synaptic receptors (Colbran and Brown, 2004; Opazo et al., 2010), and regulation of synapse proliferation and apoptosis through gene transcription (Lonze and Ginty, 2002; Wiegert and Bading, 2011). Human studies have observed BM-related cognitive improvements (Pase et al., 2012), including improved immediate (Kumar et al., 2016) and delayed memory recall (Roodenrys et al., 2002; Barbhaiya et al., 2008; Calabrese et al., 2008; Morgan and Stevens, 2010), processing speed (Stough et al., 2001), and sustained attention (Stough et al., 2008). These effects were observed after similar periods of time including 12 weeks (Stough et al., 2001; Barbhaiya et al., 2008; Calabrese et al., 2008; Morgan and Stevens, 2010), 90 days (Stough et al., 2008), and 3 months (Roodenrys et al., 2002), suggesting BM-specific improvements to cognitive outcomes might be observed after a relatively short period of supplementation. In addition, three of these studies (Stough et al., 2001, 2008; Roodenrys et al., 2002), and two acute studies (Downey et al., 2013; Benson et al., 2014) have observed effects after using a standardized form of BM called CDRI-08, which was first developed by the Central Drug Research Institute in India and is standardized to contain 55 ± 5% bacoside content (Stough et al., 2013). Bacosides have been suggested to be the main bioactive component of BM (Majumdar et al., 2013) and have shown to enhance the activity of enzymes implicated in downstream synaptogenic cellular processes (Liu et al., 2013). Using a standardized form such as CDRI-08 may, therefore, be a viable addition to CT regimes to supplement the neuroprotective properties CT provides to older adults (McPhee et al., 2016). The aim of the study was to determine whether the addition of BM (CDRI-08) to older adults regularly completing CT over 12 weeks improved cognitive and microstructural outcomes.

## Materials and Methods

### Participants

Participants were healthy, older (≥55 years), right-handed adults with no diagnosis of a psychiatric, neurological or food metabolism disorder, no history of repeated head injury or substance abuse, non-smokers, didn’t consume ≥15 standard alcoholic drinks weekly and didn’t have hypertension (≥140/90). Participants were excluded if they were taking any cognitive- or mood-altering medications or supplements, had any implanted electronically- or magnetically-activated devices, scored ≤23 on the Mini-Mental State Examination (Folstein et al., 1975) or ≥20 on the Beck Depression Inventory-II (Beck et al., 1996). The study was registered with ANZCTR (ACTRN12617001101370) and approved by the Swinburne University Research Ethics Committee (SUHREC 2017/047) in accordance with the Declaration of Helsinki and Good Clinical Practices. All participants provided written informed consent.

### Study Design

The study was a randomized, double-blind and placebo controlled for BM supplementation study. Participants were randomized to receive either BM or placebo using a computerized random number generator by a disinterested third party who was not involved in testing participants or data analysis. Treatment group allocation was also stratified by gender by the disinterested third party. All participants were required to complete CT.

Given the majority of research assessing the effects of BM in humans have demonstrated 12 weeks of BM supplementation produces cognitive effects (e.g., Roodenrys et al., 2002; Barbhaiya et al., 2008; Calabrese et al., 2008; Stough et al., 2008, 2001; Morgan and Stevens, 2010), participants were required to complete 12 weeks of the intervention. Participants attended three visits: a screening visit, baseline visit 1–2 weeks later, and a final visit 12 weeks later. During these visits blood samples were collected, questionnaires, cognitive testing, and MRI conducted. At baseline, all participants were shown how to use the CT program and were randomized to take either placebo or BM. Participants were contacted in the first 2, 4, and 8 weeks from baseline to discuss study progress and to record any significant lifestyle or health changes.

### Interventions

The active treatment was two capsules of Keenmind^TM^ (CDRI-08). Each capsule contained 160 mg of BM extract equivalent to 2.16 g of dried herb. Placebo capsules contained inactive ingredients and were matched to the treatment capsules in color and size. Participants took two capsules each morning with breakfast for 12 weeks.

All participants completed CT using the online BrainHQ^TM^ portal^1^ designed by Posit Science^TM^. Six exercises were chosen based on the original Brain Fitness program (Mahncke et al., 2006). This program has shown to be effective in multiple high-quality RCTs with older adults (Shah et al., 2017). Participants completed 2–3, 60-min sessions per week, as this has shown to be an effective dose to observe changes in cognitive performance and structural plasticity in older adults (Lampit et al., 2014, 2015).

The BrainHQ^TM^ online group portal enabled the study investigator to check the progress of each participant. CT compliance was monitored weekly at first, and then adjusted depending on the participant’s adherence to the CT dose and program. If a participant was completing less than 2–3 60 min sessions per week and/or they were consistently performing poorly, they were contacted to encourage compliance and to determine why the participant was not completing the CT as required. This contact was in addition to the routine 2, 4, and 8 week contact. Participants were asked to complete a tablet taking log throughout the 12-week period and to return any remaining tablets to calculate treatment compliance.

### Assessments

#### Cognitive and Psychological Assessments

Cognition was assessed using CogTrack^TM^, which is specially designed to be administered over more than one timepoint within a clinical trial context (Wesnes et al., 2017). It comprises of a set of 10 computerized tasks designed to assess cognitive abilities adversely affected with increasing age (Glisky, 2007; Singh-Manoux et al., 2012). The tests include remembering 15 words immediately and after a 15-min delay (immediate and delayed word recall), choosing words from the original 15 words amongst distractor words after a 15-min delay (delayed word recognition), presentation of 20 objects, and after a 15-min delay, identifying the original objects amongst similar novel objects (pattern separation), responding quickly to the presentation of a right arrow (simple reaction time, SRT), pressing a left or right arrow key when the corresponding arrow is presented (Choice Reaction Time), responding when a digit in the middle of the screen matches a target digit (digit vigilance), presentation of a 3 × 3 array of light bulbs with some light bulbs illuminated, then identifying in similar arrays what light bulbs were originally illuminated and which are novel illuminations (spatial working memory), and responding when a digit from a set of five target digits are presented amongst 30 distractor digits (numeric working memory). All participants completed a practice session during their screening visit to familiarize themselves with the cognitive tasks.

Life satisfaction was assessed using the CASP-19 scale (Hyde et al., 2003), everyday memory complaints with the prospective and retrospective memory questionnaire (Crawford et al., 2003), IQ using the Wechsler abbreviated scale of intelligence (Wechsler, 1999), and mood with the profile of moods states (McNair et al., 1992).

#### Biochemical Assessment

Brain-derived neurotrophic factor (BDNF) was used as a peripheral biomarker as it has shown to be involved in neuronal growth and maintenance (Poo, 2001; Gomez-Palacio-Schjetnan and Escobar, 2013). Blood samples were collected by venepuncture on the morning (8:30am–12:00pm) of the baseline and 12-week visit. Participants fasted for 12 h and did not perform any vigorous physical activity 24 h beforehand. Blood samples were kept at room temperature for 30 min and then centrifuged at 1,000 × *g* for 10 min. Serum was extracted, aliquoted, and stored at −80°C until analysis. Samples were sent frozen in one batch to Crux Biolabs in Melbourne, Australia for analysis. Samples were thawed and BDNF concentrations measured using the Quantibody^®^ Multiple ELISA array (RayBiotech, Norcross, GA, United States) according to the manufacturer’s instructions.

### Image Acquisition

Brain data was acquired on a 32-channel head coil equipped 3T Siemens Tim Trio using a double-refocused single-shot EPI sequence. 72 slices (2 mm^3^) were obtained in an anterior to posterior direction interleaved using an MB factor of two and two b-values: b1,000 and b2,000 s/mm^2^ with 64 diffusion-encoding gradient directions each. Four b0 images were collected, one acquired with a reversed-phase encoding direction, to allow the estimation of susceptibility induced distortions. TE and TR were matched across b-value shells (TR = 8,600 ms; TE = 99 ms).

### Imaging Data Processing

#### Diffusion Tensor Imaging

Two diffusion tensor imaging (DTI) measures, fractional anisotropy (FA) and mean diffusivity (MD), represent the degree of anisotropy of water molecules around neuroanatomical structures (Wozniak and Lim, 2006; Johansen-Berg and Behrens, 2014). They infer both the presence and health of WM with an FA decrease and an MD increase representing microstructural deterioration (Basser, 1995; Beaulieu, 2011). A detailed description of the DTI analysis is in the Supplementary Material. Briefly here, the b1,000 s/mm^2^ shell data was pre-processed (correction for susceptibility induced distortions, head movements and eddy currents) using FSL (v6.0) (Smith et al., 2004), the tensor fitted using FSL’s diffusion toolbox (FDT) (Behrens et al., 2003, 2007) to produce FA and MD maps for each timepoint, subjecting the data to a non-biased longitudinal analysis procedure (Engvig et al., 2012), in which data is non-linearly registered using Tract-based spatial statistics (TBSS) (Smith et al., 2006) to a study-specific template that represents the space halfway between the two time points, and then creating difference maps by subtracting 12-week registered maps from baseline registered maps and using these for voxel-wise comparisons. MD difference maps were produced in the same method using FSL’s non-FA TBSS procedure.

#### Neurite Orientation Dispersion and Density Imaging

Neurite orientation dispersion and density imaging (NODDI) was used to assess any intervention-related changes in the content and health of neurite microstructure. The NODDI model utilizes the movement of water molecules in and around neuronal structures to differentiate between three tissue environments: intracellular, extracellular and cerebrospinal (CSF) (Zhang et al., 2012). The intracellular environment represents the space bounded by the membrane within neurites and is modeled as a set of sticks (cylinders with zero radius), and these sticks can be highly parallel (such as in highly aligned WM structures) or highly dispersed (such as fanning or bending WM structures, or the extensive dendritic branching seen in gray matter; GM). Two NODDI parametric maps were used; orientation dispersion index (ODI), which measures the dispersion of sticks (0 = no dispersion, 1 = full dispersion) and neurite density (ND), which is the degree of neurite concentration and packing (0 = low ND, 1 = high ND).

A detailed description of the NODDI analysis is in the Supplementary Material. Briefly, data from the b1,000 and b2,000 shells were combined and pre-processed using FSL and the NODDI model fitted using the MATLAB NODDI toolbox to produce ODI and ND maps. The same non-biased longitudinal procedure in the DTI TBSS analysis was used to measure ODI and ND WM microstructure using FSLs non-FA TBSS script and difference maps created for voxel-wise analysis. GM-based spatial statistics (GBSS) was used (Nazeri et al., 2015; Supplementary Figure 1) to compare change in GM microstructure. GBSS firstly segments the GM fraction using diffusion data and then utilizes the TBSS script to accurately aligned GM voxels between subjects. This creates a GM-skeleton representing the center of GM voxels common to the group which is then used to register ODI and ND maps to each time point. Difference maps are then created by subtracting each participant’s skeletonized ODI and ND baseline maps from their corresponding 12-week skeletonized maps and used for voxel-wise comparisons.

### Statistical Analysis

#### Cognitive, Psychological and Biochemical Data

Statistical analysis was conducted using SPSS v26. Pearson’s correlation coefficients were used to identify potential covariate baseline characteristics (such as age, gender, baseline microstructure etc.) that were significantly associated with outcomes at 12 weeks. Any variables not normally distributed or showing heterogeneity of variance (Shapiro–Wilk test < 0.05 and Levene’s test < 0.05) were transformed using the Box-Cox transformation procedure (Box and Cox, 1964). Variables with severe non-normality not improved by transformations were analyzed using the non-parametric Quade Rank test (Quade, 1967). Variables with severe violations of homogeneity of variance not improved by transformations were analyzed using the Weighted Least Squares Regression method (Rosopa et al., 2013). To determine significant (α < 0.05) differences between groups from baseline to follow up, separate one-way ANCOVAs were conducted for each outcome using baseline score as a covariate (Vickers, 2005). For significant differences, separate paired sample *t*-tests for each group were then used to establish the trajectory of change within each group.

#### Diffusion-Weighted Data

Pearson’s correlation coefficient was used to identify if any baseline variables were associated with mean whole-brain WM FA, MD, ODI, ND and GM ODI, and ND at 12-weeks, and if so were controlled for in subsequent analysis. For all diffusion data voxel-wise analysis was performed using FSL’s randomize (Winkler et al., 2014). Five thousand permutations were conducted for each contrast with the threshold-free-cluster-enhancement (TFCE) option (Smith and Nichols, 2009) and family-wise error (FWE) rate correction for multiple comparisons (*p* < 0.05). To determine significant group differences in the change in DTI and NODDI metrics from baseline to 12-weeks, an unpaired samples *t*-test was conducted using the TBSS generated FA, MD, ODI, and ND and the GBSS generated ODI and ND difference maps. Mean change within significant clusters were extracted for each group to determine the direction of change. The John Hopkins University (JHU) ICBM-DTI-81 White-Matter Labels atlas (Mori et al., 2008), the JHU White Matter Tractography Atlas (Hua et al., 2008) and the Harvard-Oxford Cortical Atlas (Desikan et al., 2006) were used to identify significant brain regions. Given the relatively novel and under-researched intervention in this study, uncorrected maps were also searched for significant voxels *p* < 0.005 within cluster sizes of ≥10 (Lieberman and Cunningham, 2009), to determine trends within the neuroimaging data. To see if any clusters were related to any significant cognitive outcomes, a step-wise regression was performed for each group using mean change extracted from each cluster as the IV and change in cognitive score as the DV. The criteria used for each step were based on *F*-tests, with entry set at *p* = 0.05 and removal set at *p* = 0.10.

## Results

### Baseline Characteristics and Rates of Follow Up

**FIGURE 1:**
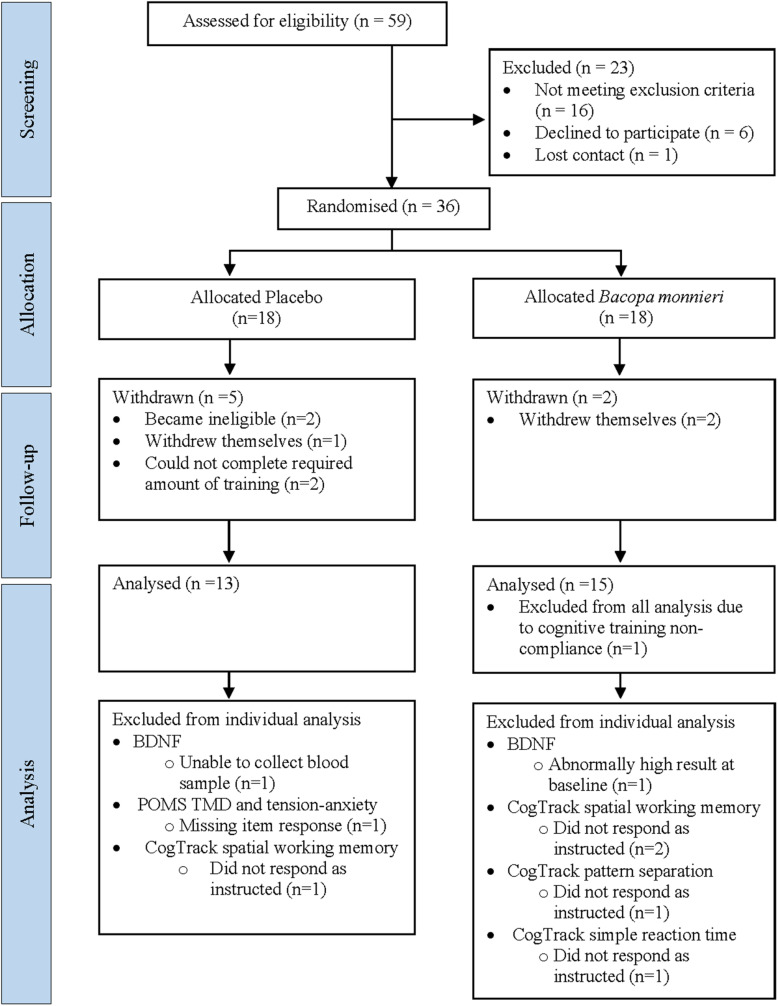
Participant enrolment flowchart. BDNF, brain-derived neurotrophic factor; POMS, profile of mood states; TMD, total mood disturbance.

Thirty-six participants were randomized to the active (*n* = 18) and placebo (*n* = 18) groups. Seven withdrew before follow-up, one was excluded from all analysis due to CT non-compliance and seven excluded from specific analyses (see Figure 1). The final sample size consisted of BM group (*n* = 15) and placebo group (*n* = 13) (age range = 57–78 years) (Table 1).

**TABLE 1:** Baseline characteristics.

	Placebo (*n* = 13)	BM (*n* = 15)
Age, mean ± SD (years)	66.85 ± 5.93	68.87 ± 5.59
Female, n (%)	8 (61.5)	8 (53.3)
Education, mean ± SD (years)	15.23 ± 3.68	15.27 ± 3.41
Employment status, n (%)		
Part time/casual	4 (30.8)	4 (26.7)
Full time	2 (15.4)	1 (6.7)
Retired	7 (53.8)	10 (66.7)
IQ, mean ± SD (average IQ = 100)	126.92 ± 8.75	123.33 ± 11.28
MMSE, mean ± SD (0–30)	29.15 ± 1.14	29.00 ± 1.20
BDI, mean ± SD (0–63)	3.31 ± 4.97	2.27 ± 2.84
Total hours trained, mean ± SD	36.40 ± 4.79	36.18 ± 6.38
Treatment compliance, mean ± SD (%)	99.09 ± 1.57	98.59 ± 5.13
Blood draw time, mean ± SD (hh:mm)	08:36 ± 00:39	09:27 ± 01:22
Whole-brain DWI, mean ± SD		
WM FA (0–1)	0.484 ± 0.025	0.476 ± 0.018
WM MD (10^–3^ mm^2^/s)	0.741 ± 0.036	0.751 ± 0.035
WM ODI (0–1)	0.270 ± 0.023	0.271 ± 0.024
WM ND (0–1)	0.609 ± 0.031	0.603 ± 0.030
GM ODI (0–1)	0.535 ± 0.006	0.527 ± 0.030
GM ND (0–1)	0.449 ± 0.013	0.448 ± 0.013

*IQ,Wechsler adult scale of intelligence; MMSE, Mini Mental State Examination; BDI, Beck depression inventory; WM, white matter; GM, gray matter; FA, fractional anisotropy; MD, mean diffusivity; ODI, orientation dispersion index; ND, neurite density.*

### Cognitive, Psychological, and Biochemical Outcomes

**FIGURE 2:**
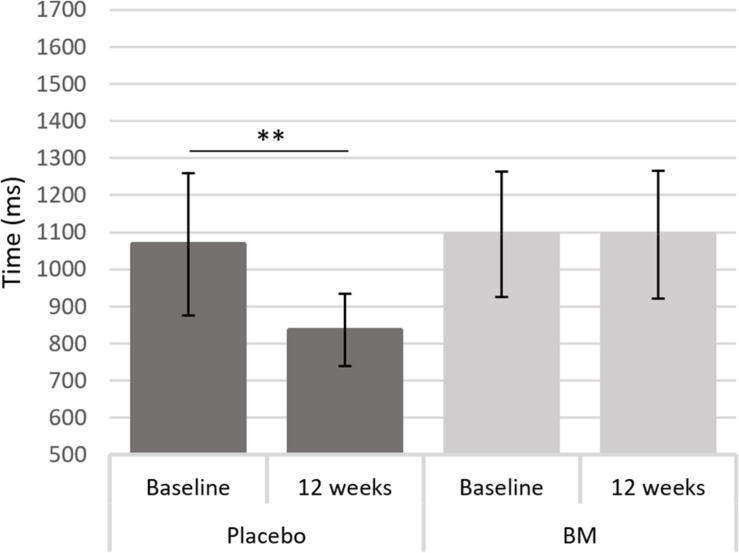
Difference in reaction time to the original stimulus in the spatial working memory task from baseline to follow up in the placebo and BM group. ^∗∗^< 0.005.

**FIGURE 3:**
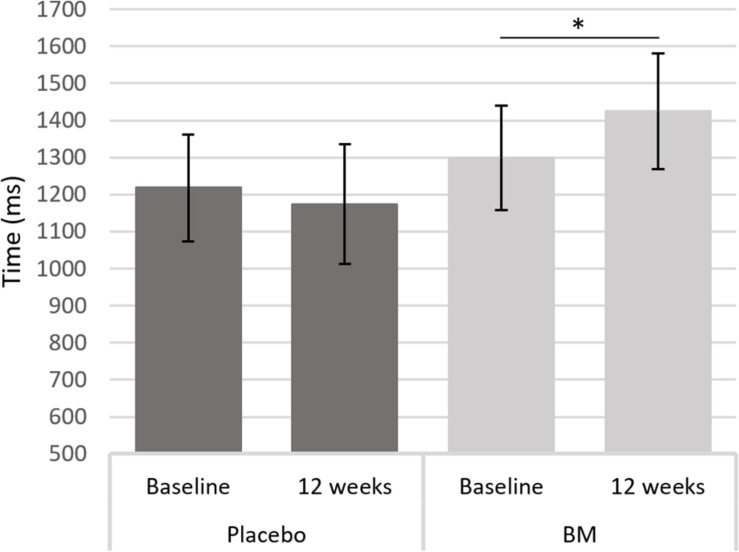
Difference in reaction time to a novel stimulus in the pattern separation task from baseline to follow up in the placebo and BM group. ^∗^< 0.05.

One-way ANCOVAs showed there was a significant difference in speed between groups at follow up after controlling for baseline scores for SRT, for remembering novel images from a set of similar images in the pattern separation task (PS-N RT), and for remembering original lightbulb configurations in the spatial working memory task (SWM-O RT) (Table 2). Follow up *t*-tests showed there was a significant decrease in SWM-O RT in the placebo group [*t*(11) = −3.604, *p* = 0.004] and no significant change in the BM group [*t*(12) = −0.002, *p* = 0.999; Figure 2]; no significant change in PS-N RT in the placebo group [*t*(11) = −0.934, *p* = 0.370] and a significant increase in the BM group [*t*(14) = 2.780, *p* = 0.015; Figure 3]; and no significant change in SRT in neither the placebo or BM group [*t*(12) = −1.158, *p* = 0.269; *t*(13) = 1.994, *p* = 0.068, respectively].

**TABLE 2:** 12 weeks follow-up scores for psychological, cognitive and biochemical outcomes after adjustment for baseline score.

Outcome	*N*	Unadjusted means (95% CI)			Adjusted means (95% CI)		ANCOVA	
		Placebo	BM	Covariate	Placebo	BM	*F* (df)	*P*
**CogTrack**								
**Immediate word recall**								
Accuracy (%)	28	33.7 (28.8, 37.6)^*a*^	33.4 (27.8, 37.8)^*a*^		32.8 (27.6, 36.8)^*a*^	34.2 (29.9, 37.8)^*a*^	0.25 (1,25)	0.619
**Simple reaction time**								
Speed (ms)	27	346.7 (327.9, 365.6)	390.9 (359.8, 422.0)		350.4 (331.5, 369.4)	387.4 (369.2, 405.7)	8.36 (1,24)	**0.008**
**Digit vigilance**								
Accuracy (%)	28	98.8 (97.4, 99.9)^*a*^	96.8 (94.5, 98.4)^*a*^	Age	98.5 (97.2, 99.5)^*a*^	97.1 (95.6, 98.3)^*a*^	2.61 (1,24)	0.119
Speed (ms)	28	484.2 (466.7, 501.8)	505.5 (477.8, 533.2)		499.1 (479.8, 519.3)	492.6 (473.9, 511.3)	0.21 (1,25)	0.654
**Choice reaction time**								
Accuracy (%)^*b*^	28	98.0 (96.5, 99.3)^*a*^	97.0 (95.4, 98.3)^*a*^	Education	97.6 (96.3, 98.7)^*a*^	97.4 (96.2, 98.4)^*a*^	0.05 (1,24)	0.828
Accuracy (%)^*b*^	28	98.1 (96.7, 99.3)^*a*^	97.1 (95.6, 98.3)^*a*^	WM MD	97.6 (96.3, 98.7)^*a*^	97.3 (96.3, 98.6)^*a*^	0.02 (1,24)	0.903
Speed (ms)	28	517.9 (484.7, 551.1)	560.6 (531.0, 590.3)		528.3 (511.3, 545.4)	551.6 (535.8, 567.4)	4.16 (1,25)	0.052
**Spatial working memory**								
Original stimuli accuracy (%)	25	95.8 (91.9, 99.7)	97.6 (94.0, 101.2)	Age	96.1 (92.6, 99.5)	97.4 (94.1, 100.7)	1.06 (1,23)	0.315^*c*^
Novel stimuli accuracy (%)	25	94.2 (90.1, 98.2)	96.5 (93.4, 99.7)		94.2 (90.8, 97.7)	96.5 (93.1, 99.8)	0.93 (1,23)	0.345^*c*^
Original stimuli speed (ms)	25	816.7 (701.0, 948.2)^*a*^	1066.0 (880.0, 1273.5)^*a*^	IQ	847.2 (741.7, 964.1)^*a*^	1032.2 (914.4, 1158.7)^*a*^	5.16 (1.21)	**0.034**
Novel stimuli speed (ms)	25	964.3 (796.8, 1131.9)	1114.0 (908.9, 1319.1)	IQ	974.5 (819.3, 1129.7)	1104.7 (955.9, 1253.4)	1.33 (1,23)	0.261^*c*^
**Numeric working memory**								
Original stimuli accuracy (%)	28	92.9 (87.3, 98.4)	98.7 (96.6, 100.7)		92.7 (86.2, 99.3)	98.9 (97.3, 100.5)	3.55 (1,25)	0.071^*d*^
Novel stimuli accuracy (%)	28	99.0 (96.7, 101.2)	99.6 (98.6, 100.5)		99.0 (97.4, 100.6)	99.5 (98.0, 101.1)	0.08 (1,26)	0.787^*c*^
Original stimuli speed (ms)	28	781.7 (675.3, 888.1)	861.4 (788.5, 934.4)		819.7 (776.5, 862.9)	828.5 (788.3, 868.6)	0.09 (1,25)	0.766
Novel stimuli speed (ms)	28	987.8 (718.1, 1125.2)^*a*^	938.7 (808.7, 1088.2)^*a*^		980.9 (857.4, 1120.2)^*a*^	866.9 (766.2, 982.0)^*a*^	1.80 (1,25)	0.191
**Delayed word recall**								
Accuracy (%)	28	27.7 (20.7, 36.6)	24.9 (17.6, 32.2)		26.7 (20.5, 33.0)	25.7 (19.9, 31.6)	0.05 (1,25)	0.818
**Word recognition**								
Original stimuli accuracy (%)	28	75.4 (67.3, 83.5)	71.6 (62.4, 80.8)	Hours trained	78.1 (70.3, 85.9)	69.2 (62.0, 76.4)	2.74 (1,24)	0.111
Novel stimuli accuracy (%)	28	89.7 (83.3, 96.2)	91.1 (85.6, 96.6)		88.9 (83.6, 94.2)	91.9 (86.9, 96.8)	0.71 (1,25)	0.408
Original stimuli speed (ms)	28	845.6 (755.1, 1003.0)^*a*^	914.7 (835.8, 1018.0)^*a*^		880.9 (796.7, 1001.0)^*a*^	869.9 (794.3, 972.9)^*a*^	0.03 (1,25)	0.876
Novel stimuli speed (ms)	28	950.4 (852.4, 1048.3)	1017.3 (903.9, 1130.7)	IQ	998.5 (916.4, 1080.7)	975.5 (899.4, 1051.6)	0.17 (1,24)	0.685
**Pattern separation**								
Original stimuli accuracy (%)	27	90.8 (85.8, 95.9)	90.3 (86.1, 94.6)		90.8 (86.0, 95.5)	90.4 (86.2, 94.6)	0.01 (1,24)	0.910
Novel stimuli accuracy (%)	27	76.6 (66.5, 84.4)^*a*^	80.0 (74.1, 85.1)^*a*^		74.7 (66.9, 81.0)^*a*^	81.4 (75.9, 86.1)^*a*^	2.63 (1,24)	0.118
Original stimuli speed (ms)	27	1134.0 (960.2, 1402.1)^*a*^	1146.9 (1049.4, 1266.6)^*a*^		1126.0 (1030.2, 1244.8)^*a*^	1141.7 (1052.1, 1249.9)^*a*^	0.05 (1,24)	0.824
Novel stimuli speed (ms)	27	1108.3 (970.3, 1307.4)^*a*^	1384.4 (1227.9, 1569.5)^*a*^		1141.1 (1059.7, 1237.2)^*a*^	1340.8 (1242.0, 1451.2)^*a*^	8.79 (1,23)	**0.007**
**PRMQ**								
Prospective memory (8–40)	28	31.2 (28.9, 33.6)	29.8 (27.3, 32.3)	GM ND	30.7 (29.3, 32.1)	30.3 (28.9, 31.6)	0.19 (1,24)	0.668
Retrospective memory (8–40)	28	31.7 (29.2, 34.2)	30.5 (28.0, 33.0)		31.3 (29.8, 32.8)	30.8 (29.4, 32.2)	0.29 (1,25)	0.594
**CASP19**								
Control (0–18)	28	14.5 (13.1, 16.0)	13.5 (11.8, 15.1)	Gender	14.1 (12.7, 15.5)	13.9 (12.6, 15.2)	0.34 (1,24)	0.568
Autonomy (0–15)	28	12.8 (11.8, 13.8)	11.9 (10.5, 13.2)		12.3 (11.2, 13.4)	12.3 (11.2, 13.3)	0.01 (1,25)	0.921
Self-realization (0–12)	28	10.4 (9.5, 11.2)^*a*^	8.7 (7.2, 10.0)^*a*^		10.0 (9.0, 10.9)^*a*^	9.2 (8.2, 10.1)^*a*^	1.32 (1,25)	0.262
Pleasure (0–12)	28	11.1 (10.1, 11.9)^*a*^	10.3 (9.2, 11.2)^*a*^		10.8 (10.2, 11.4)^*a*^	10.6 (10.0, 11.1)^*a*^	0.39 (1,25)	0.539
Total (0–57)	28	48.2 (45.3, 51.2)	43.3 (38.0, 48.6)		45.3 (42.2, 48.5)	45.8 (42.8, 48.7)	0.04 (1,25)	0.847
**POMS**								
Anger-hostility (0–48)	28	0.9 (−0.1, 1.8)	2.8 (1.1, 4.5)		1.6 (0.6, 2.6)	2.2 (1.2, 3.1)	0.29 (1,26)	0.596^*c*^
Confusion-bewilderment (0–28)	28	4.5 (3.8, 5.4)^*a*^	6.9 (5.1, 9.8)^*a*^		4.5 (3.8, 5.6)^*a*^	4.9 (4.1, 6.0)^*a*^	0.41 (1,25)	0.527
Depression-dejection (0–60)	28	1.7 (0.8, 2.9)^*a*^	4.2 (2.0, 7.5)^*a*^		2.1 (1.1, 3.4)^*a*^	2.5 (1.5, 3.8)^*a*^	0.30 (1,25)	0.590
Fatigue-inertia (0–28)	28	4.2 (2.4, 6.1)	4.7 (2.8, 6.7)	GM ODI	4.7 (3.3, 6.0)	4.4 (3.1, 5.2)	0.10 (1,24)	0.759
Tension-anxiety (0–36)	27	4.3 (3.3, 5.4)	6.5 (4.5, 8.5)		5.0 (3.5, 6.6)	5.9 (4.5, 7.3)	0.73 (1,24)	0.402
Vigor-activity (0–32)	28	19.5 (16.3, 22.8)	18.8 (15.5, 22.1)		17.9 (15.6, 20.3)	20.2 (18.0, 22.4)	2.04 (1,25)	0.166
TMD (−32–200)	27	−4.8 (−9.7, 0.0)	6.1 (−3.8, 16.0)		0.1 (−1.8, 2.1)	6.0 (−1.6, 13.5)	2.38 (1,24)	0.136^*d*^
**BDNF (pg/ml)**	26	2935.9 (2156.3, 3715.4)	3264.8 (2088.7, 4440.9)		2954.9 (2433.9, 3475.9)	3245.7 (274.8, 3766.7)	0.67 (1,23)	0.423

*WM MD, white matter mean diffusivity at baseline; PRMQ, prospective and retrospective memory questionnaire; GM ND, gray matter neurite density at baseline; POMS, profile of mood states; GM ODI, gray matter orientation dispersion index at baseline; BDNF, brain-derived neurotrophic factor; TMD, total mood disturbance.*

*^*a*^Back transformed from Box-Cox transformation.*

*^*b*^Covariates correlated with each other so two separate models for each covariate tested.*

*^*c*^Quade-rank test.*

*^*d*^Weighted least squares regression. Bold values are to highlight that they are statistically significant.*

### Neuroimaging Outcomes

#### DTI

Unpaired sample *t*-tests showed there were no significant differences in FA and MD change between groups after correction for multiple comparisons. Uncorrected analysis (Supplementary Table 1) showed significant groups differences in three clusters for WM FA, and in 28 clusters for WM MD. Means extracted from these clusters showed either an increase or a less severe decline in FA and a decrease or a less severe increase in MD in the placebo group compared to the BM group. For the step-wise regression (Supplementary Table 1) in the placebo group, the best model to predict a decrease (improvement) in SWM-O RT was an increase in MD in the right corticospinal tract, right anterior longitudinal fasciculus and an area near the superior parietal lobule and a decrease in MD in the right anterior thalamic radiation. For the BM group, the best model to predict a decrease (improvement) in SWM-O RT was an increase in MD in the right optic radiation and a decrease in MD in the right forceps major. No clusters showing differences in FA predicted change in SWM-O RT nor for MD and FA in significantly predicting change in PS-N RT.

#### NODDI

Unpaired samples *t*-test showed there was no significant difference in ODI or ND change in GM and WM between groups after correction for multiple comparisons. Uncorrected analysis (Supplementary Table 2) showed four clusters showing ND increases in the placebo group and decreases in the BM group, and one cluster showing a decrease in the placebo group and an increase in the BM group. Ten clusters showed ODI decreases in the placebo group and increases in the BM group, with two clusters showing an increase in the placebo group and a decrease in the BM group. Step-wise regression (Supplementary Table 2) in the placebo group showed a decrease in GM ODI in the left putamen and a decrease in GM ND in the right planum polare significantly predicted a decrease (improvement) in SWM-O RT. In the BM group, an increase in ODI in the left frontal orbital cortex significantly predicted a decrease (improvement) in SWM-O RT. No cluster for ODI nor ND significantly predicted change in PS-N RT.

## Discussion

The aim of the current study was to determine if BM is an effective supplement to CT to improve cognitive and neuroimaging outcomes in an older sample. The overall results were mixed. The results showed RT increased in the BM group in the PS-N task, with no change in the placebo group, and RT decreased in the SWN-O task in the placebo group with no change in the BM group. Although group comparisons for accuracy did not reach statistical significance, examination of the means suggest the BM group were more accurate in the PS-N task than the placebo group despite their slower response times (see Table 2). This may reflect a speed-accuracy trade-off in which the BM group favored precision over speed. Previous research suggests BM supplementation increases accuracy in tasks measuring learning and free recall memory (Pase et al., 2012) and has been used traditionally as a memory enhancer in Ayurvedic medicine (Russo and Borrelli, 2005). This suggests BM may benefit accuracy specifically and not alter speed. Despite there being an apparent difference in the means in accuracy scores, it is important to note that these differences were not statistically significant and therefore only inferences can be made about the inter-relationship between accuracy and speed in the current study.

There were also differences at baseline that may have disadvantaged the BM group to perform faster. Previous research has shown older adults favor minimizing errors over speed compared to younger adults, even when they are cued to respond quickly (Starns and Ratcliff, 2010, 2012) and this may be due to age-related limitations in brain structure (Forstmann et al., 2011; Yang et al., 2015). The placebo group was slightly younger than the BM group and had slightly better brain microstructure at baseline (higher FA and GM ODI and lower MD). The placebo group also had higher IQs and trained an average of 13 min more than the BM group. These differences combined may have contributed to the slower PS-N RT in the BM group and faster SWM-O RT in the placebo group. Consequently, BM supplementation may not have produced cognitive improvements large enough to reliably detect over and above that of a younger neurocognitively healthier group who were also benefiting from more CT. Age, baseline microstructure, IQ and hours trained were tested for inclusion as covariates in the analyses, and none were associated with PS-N RT at 12 weeks and the effects on SWM-O RT did not change after controlling for IQ. This indicates there may have been other influencing, unaccounted for factors on the cognitive outcomes or BM supplementation does not impact RT above the effects of CT alone.

The other interpretation is that BM may impair processing speed for visuospatial memory. This effect has not been observed in other research however, as studies using similar tests have not observed any changes to speed [e.g., no change to speed in SWM-O RT in Stough et al. (2008), or to visual memory span in Roodenrys et al. (2002) and Barbhaiya et al. (2008)]. It is therefore difficult to substantiate if this is a replicable effect, or spurious due to a small sample size.

The neuroimaging results did not provide a compelling neuro-structural explanation as there were no differences in microstructure change between groups after correction for multiple comparisons. Given the sample size was small, neuroimaging of BM is relatively under-researched, and both groups were completing an intervention (CT) concurrently, the effects on microstructure from BM alone were speculated to be very slight. As such, exploratory analysis, uncorrected for multiple comparisons at a stricter p-value (*p* < 0.005), were utilized to identify small effects (Lieberman and Cunningham, 2009). The uncorrected results produced clusters showing group differences largely categorized into one directional effect (Supplementary Table 1). This may demonstrate the results were not necessarily spurious and taking BM may have produced some small but homogeneous effects over time. For DTI there was a decrease in WM FA and increase in WM MD in the BM group and the opposite pattern in the placebo group. This suggests either WM microstructure improved in the placebo group or it declined in the BM group. These unexpected results may, again, be occurring by chance due to the small sample size. They may also be indicative of a poorly defined measure of WM structure. MD and FA are based on assumptions of water movement around cellular structures, with more free water movement implying an increase in tissue breakdown (Wozniak and Lim, 2006; Beaulieu, 2011). DTI has been validated against cases of tissue degeneration such as from multiple sclerosis (Bammer et al., 2000; Cercignani et al., 2001; Ciccarelli et al., 2001), Wallerian disease (Pierpaoli, 2010), Alzheimer’s disease (Friese et al., 2010; Dyrba et al., 2013), and cerebral ischemia (Sorensen et al., 1999). Consequently, it may be indicative of clinical neurodegeneration, but less so in the context of neuroplasticity in non-clinical samples. Research has observed measurable benefits of learning with increased MD and decreased FA, including an association between a training-related improvement in working memory task performance with an increase in MD (Takeuchi et al., 2015), increased MD and decreased FA after training of a complex balancing task (Taubert et al., 2010) and increased free water movement with an increase in neural activity (Song et al., 2002). Bilinguals, who have undergone long-term neuroplastic changes to learn another language, have shown higher MD and lower FA compared to monolinguals (Cummine and Boliek, 2013; Singh et al., 2018). In these cases, increases in MD and decreases in FA may be indicative of an increase in axon diameter, potentially from the development of additional crossing fiber bundles (as suggested in Zatorre et al., 2012). Unfortunately, complex tissue architecture such as crossing, or bending fibers that deviate from the parallel, coherent fibers assumed in the DTI model, can make interpretation of DTI outcomes challenging (Jones et al., 2013). This was also evident in the step-wise regression analyses, in which some clusters associated with SWM-O RT showed a negative relationship and others a positive relationship with MD change. As such the assumed model DTI is based on might not be adequately capturing minute structural changes in response to learning in a small non-clinical sample.

Neurite orientation dispersion and density imaging uses a multi-compartment model that better captures intricate cell architecture (Zhang et al., 2012). Exploratory analysis showed there was an ND decrease and an ODI increase in the BM group (Supplementary Table 2). An increase in ODI indicates an increase in the complexity of the GM neuropil such as extensive branching of dendritic trees (Zhang et al., 2012). High ODI has been associated with evidence of neuroplasticity within the human cortex, including an association with high functional connectivity within the default mode network (Nazeri et al., 2015) and is predictive of inter-hemispheric functional connectivity (Deligianni et al., 2016). The observed decrease in ND may demonstrate experience-dependent restructuring of cytoarchitecture such as dendritic pruning (Riccomagno and Kolodkin, 2015). Research on brain maturation shows substantial brain reorganization, which includes both propagation and pruning of dendritic pathways (Huttenlocher, 1990). Animal research has observed mice trained in a sensory or navigation task show both dendritic spine arborization and pruning (Knafo et al., 2005; Hawes et al., 2015). Research using connectivity models suggests a higher number of axons increases the neuropil volume and reduces neuronal efficiency, while a larger number of morphological features such as axonal and dendritic branching or spines, reduces volume and simultaneously increases efficiency (Chklovskii, 2004). An increase in network complexity (as seen by the increase in ODI) with a simultaneous reduction in the density and packing of neurites in the BM group, may therefore be indicating a training-induced and BM enhanced optimization of functional networks.

Other psychometric and biomarker measures failed to show significant group differences. Given the relatively healthy sample, that all participants completed a cognitively enhancing intervention (CT), and therefore gain benefits regardless, and the purported BM associated synaptogenic mechanisms are based on microscopic molecular changes within animal tissue, the effects of BM may have been very small. Limitations in the study may have impeded the ability to capture these small changes. For example, using Region of Interest (ROI) techniques instead of whole-brain analysis may have better captured smaller region-based effects. Whole-brain analysis was used as there is currently no neuroimaging studies assessing the effects of BM that may have helped inform potential *a priori* regions of interest. The intervention length could have been longer to show larger effects. The 12-week timeframe was chosen as it is what most BM studies showing cognitive effects have used (Pase et al., 2012) and it seemed a reasonable amount of time to ask participants to complete a fairly intensive CT regime (3 h weekly) without causing fatigue or boredom [as noted in Lampit et al. (2014)]. In addition, other indicators of health that may have influenced the bioavailability and metabolism of BM, such as diet, blood pressure, and tests for glucose and lipid metabolism were not included in the study. Research has demonstrated BM may provide benefits to blood lipid profiles (Kala et al., 2015; Kumar et al., 2016) reduce blood pressure (Kala et al., 2015) and when taken with other antioxidants and phytochemicals improves scores in measures of sustained attention and verbal fluency (Crosta et al., 2021). Including these measures may have helped elucidate the combined effects of cardiovascular health, diet, and BM supplementation on measures of cognition and brain health. The scan quality could have also been improved by interspersing additional non-diffusion-weighted images between runs or capturing b-shells in both phase-encoding directions to better correct geometric distortions and signal pile-up (Pierpaoli, 2010). These were not employed due to time constraints within the scanner. A larger voxel size to improve SNR and the longitudinal stability between scan time points (Zhan et al., 2013) was also considered but not implemented as it would increase partial volume effects (Alexander et al., 2001). The type of longitudinal analysis, in which scans were registered to the half-way point, was also utilized to improve longitudinal stability and prevent artificially large changes seen when aligning the scans to only one timepoint (Thomas et al., 2009). Despite these considerations, there were still non-significant outcomes. In addition, particular cognitive data were excluded due to some participants not completing certain cognitive tasks as instructed, reducing the sample further. This data could have been included in an intention to treat analysis, however, it would unlikely provide further information over and above the per-protocol analysis, given the scores of these tests were ultimately redundant and would produce inconclusive outcomes in an already small sample.

As discussed above, intrinsic differences between groups may have affected how groups changed over time. With a larger sample, more baseline covariates that may have impacted outcomes could have been controlled for Kahan et al. (2014). Although the neuroimaging outcomes were suggestive of an improvement in the complexity of neuronal networks in older adults after BM supplementation and CT, a larger study over a longer follow-up period with a more rigorous scanning procedure and addition of statistical covariates, would help clarify how BM may change microstructural and synaptogenic biomarkers, and what benefit that may have for older adults completing CT.

## Data Availability Statement

The raw data supporting the conclusions of this article will be made available by the authors, without undue reservation.

## Ethics Statement

The studies involving human participants were reviewed and approved by the Swinburne University Research Ethics Committee. The patients/participants provided their written informed consent to participate in this study.

## Author Contributions

CS: conceptualization. GM, CS, LD, and KW: methodology. GM: analysis, investigation, data curation, and writing–original draft preparation. LD and CS: supervision. CS and LD: funding acquisition. All authors contributed to the writing–review and editing of the manuscript.

## Conflict of Interest

KW was employed by the company Wesnes Cognition Ltd. The remaining authors declare that the research was conducted in the absence of any commercial or financial relationships that could be construed as a potential conflict of interest.

## Footnotes

1 https://www.brainhq.com/
