# Supplementary material for: The Neurocognitive Effects of Bacopa monnieri and Cognitive Training on Markers of Brain Microstructure in Healthy Older Adults
Source: Front Aging Neurosci. 2021 Feb 22;13:638109. doi: 10.3389/fnagi.2021.638109 (PMC7937913; doi:10.3389/fnagi.2021.638109)
Supplement: Supplementary file 1 [file Data_Sheet_1.docx]

Supplementary Material

# Description of non-biased longitudinal diffusion tensor imaging analysis.

Using the b1000 s/mm^2^ shell, data were first corrected for susceptibility induced distortions, head movements and eddy currents and brain masks created by extracting brain tissue from the non-diffusion weighted volumes using FSL (v6.0) [1]. FA and MD maps were then generated by fitting the diffusion tensor to the data using FSL’s Diffusion Toolbox (FDT) [2,3]. Baseline FA maps were registered to 12-week FA maps, and vice versa using FSL’s SIENA script [4]. The SIENA generated halfway matrix was used to resample baseline FA maps to the halfway space between baseline and 12-week (baseline halfway maps) and vice versa (12-week halfway maps). These two half-way FA maps were then averaged using fslmaths to create a participant-specific template (base FA map). All participant base FA maps were then subjected to the following Tract-based spatial statistics (TBSS) [5] procedure: 1) non-linear registration to FMRIB58_FA standard space, 2) affline-alignment to 1mm^3^ MNI152_T1 space, 3) averaging of normalised maps to create mean FA maps of all participants, and 4) skeletonisation of mean FA maps to restrict data analyses to voxels with an FA > 0.2, generating a mean FA skeleton representing the centre of all tracts common to the group. This procedure created a study-specific FA map that was used as the study-specific template. The same TBSS procedures were applied to baseline halfway maps and 12-week halfway maps, but were non-linearly registered to the study-specific template. This produced 4D skeletonisation maps, which were then separated using fslsplit to produce individual participant skeletonised maps for both halfway baseline and 12-week maps. Changes in FA were calculated by subtracting each participant’s baseline halfway skeletonised maps to their 12-week halfway skeletonised maps using fslmaths. The same procedure was conducted for MD maps, including halfway registration and non-linearly registration to the study-specific template using FSL’s non-FA TBSS procedure, and creation of difference maps. These difference maps were used for voxel-wise comparisons.

# NODDI analysis.

First, both the b1000 and b2000 s/mm^2^ shells were combined and corrected for susceptibility induced distortions, head movements and eddy currents with FSL. Only a small number of b0 images were acquired in the scanning protocol, which can reduce the accuracy at which the b0 intensity is estimated and therefore lead to non-positive b0 values, particularly around the edge of the brain. The brain mask was therefore defined by thresholding any voxels with the b0 intensity lower than one-third of the average b0 intensity in the brain tissue. The NODDI output was generated using the MATLAB NODDI toolbox.

The same procedures in the DTI TBSS analysis were used to measure WM microstructure, in which halfway ODI and ND maps were non-linearly registered to the study-specific template using FSLs non-FA TBSS script and difference maps (between timepoints) created for voxel-wise analysis.

GBSS analysis (Figure S1) on NODDI data were conducted by 1) estimating the WM fraction using Atropos two-tissue class segmentation of the FA images [6]; 2) then subtracting the WM fraction and the NODDI produced CSF fraction from 1 in each voxel in order to estimate the GM fraction; 3) creation of ‘pseudo-T1’ images by multiplying each tissue fraction by their corresponding contrast (0 for CSF, 1 for GM and 2 for WM) and summing together; 4) group-wise nonlinear registration with the pseudo-T1 images using ANTs [7,8] to generate a study-specific template; 5) warping of GM fraction, ODI and ND images on to the study-specific template; 6) averaging and thinning of aligned GM fraction maps to produce a GM-skeleton representing the centre of GM voxels common to the group; 7) projection of each participant’s aligned ODI, ND and GM fraction images onto the skeleton, with the skeleton only retaining GM voxels >0.65 in >75% of participants; 8) filling of non-satisfactory voxels (<0.65) with the average of the surrounding satisfactory voxels on the skeleton with a Guassian kernel (default σ=2 mm). Difference maps were then created by subtracting each participant’s skeletonised ODI and ND baseline maps from their corresponding 12-week skeletonised maps. These were used for voxel-wise comparisons.

References

1. Smith SM, Jenkinson M, Woolrich MW, Beckmann CF, Behrens TEJ, Johansen-Berg H, et al. Advances in functional and structural MR image analysis and implementation as FSL. Neuroimage. 2004;23:S208–S219.

2. Behrens TEJ, Woolrich MW, Jenkinson M, Johansen-Berg H, Nunes RG, Clare S, et al. Characterization and propagation of uncertainty in diffusion-weighted MR imaging. Magn Reson Med. 2003;50:1077–1088.

3. Behrens TEJ, Berg HJ, Jbabdi S, Rushworth MFS, Woolrich MW. Probabilistic diffusion tractography with multiple fibre orientations: What can we gain? Neuroimage. 2007;34:144–155.

4. Smith SM, Zhang Y, Jenkinson M, Chen J, Matthews PM, Federico A, et al. Accurate, robust, and automated longitudinal and cross-sectional brain change analysis. Neuroimage. 2002;17:479–489.

5. Smith SM, Jenkinson M, Johansen-Berg H, Rueckert D, Nichols TE, Mackay CE, et al. Tract-based spatial statistics: Voxelwise analysis of multi-subject diffusion data. Neuroimage. 2006;31:1487–1505.

6. Avants BB, Tustison NJ, Wu J, Cook PA, Gee JC. An open source multivariate framework for n-tissue segmentation with evaluation on public data. Neuroinformatics. 2011;9:381–400.

7. Avants BB, Tustison NJ, Song G, Cook PA, Klein A, Gee JC. A reproducible evaluation of ANTs similarity metric performance in brain image registration. Neuroimage. 2011;54:2033–2044.

8. Avants BB, Yushkevich P, Pluta J, Minkoff D, Korczykowski M, Detre J, et al. The optimal template effect in hippocampus studies of diseased populations. Neuroimage. 2010;49:2457–2466.


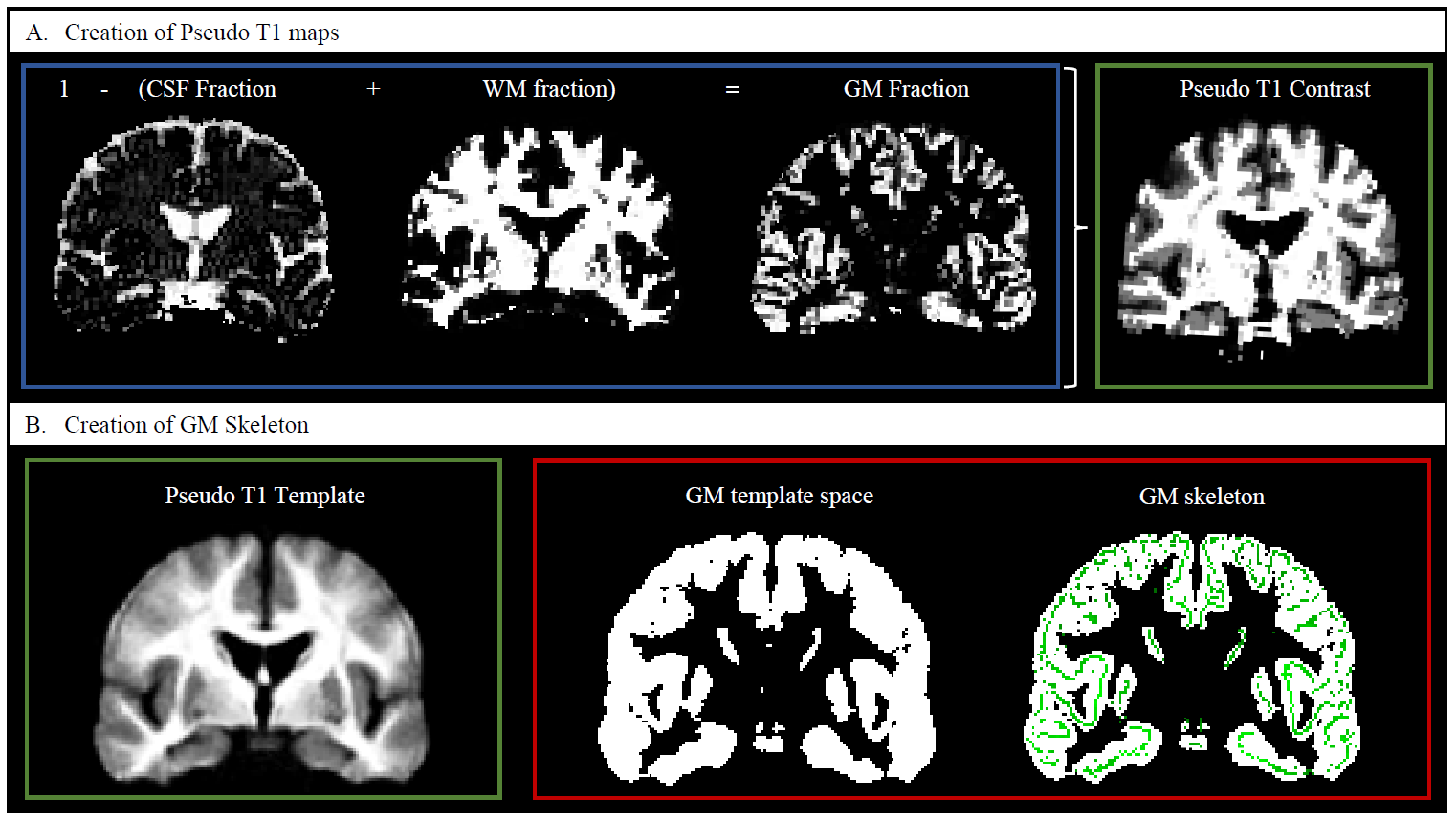


**Supplementary Figure 1*.*** (**A)** Each participant’s FA image from DTI was used to generate WM fraction maps and their CSF maps from NODDI were used to create CSF fraction maps. These were then added and subtracted from 1 to estimate the GM fraction. Appropriate contrasts were applied for each tissue fraction map and then summed to create Pseudo T1 maps. (**B)** Each pseudo T1 map were group-wise, non-linearly registered to create a study specific pseudo T1 template. GM fraction was warped to this template and then the GM fraction averaged and thinned to create a GM skeleton representing the GM voxels common to the group.

| **Supplementary Table 1.** Uncorrected effects (*p* < .005) for measures of white matter microstructure | | | | | | | | | | | |
| --- | --- | --- | --- | --- | --- | --- | --- | --- | --- | --- | --- |
|  |  |  | Local maximum | | |  |  | Mean change in clusters | | Step-wise regression *β*^±^ | |
| Measure | Region | Voxels | x | y | z | Max 1-p | T-value | Placebo | BM | Placebo | BM |
| **WM FA**^a^ |  |  |  |  |  |  |  |  |  |  |  |
| Placebo > BM | R cingulum | 31 | 25 | -46 | -2 | 0.003 | 1.23 | -0.01064 | -0.04470 |  |  |
|  | Unclassified | 15 | -36 | 15 | -5 | 0.001 | 3.16 | 0.07124 | 0.00866 |  |  |
|  | L inferior fronto-occipital fasciculus | 11 | -23 | -90 | -6 | 0.003 | 0.64 | -0.19560 | -0.25705 |  |  |
| **WM MD**^b^ |  |  |  |  |  |  |  |  |  |  |  |
| Placebo < BM | R superior longitudinal fasciculus | 151 | 48 | -34 | 11 | 0.001 | 0.90 | -0.00006 | 0.00001 |  |  |
|  | R inferior cerebellar peduncle | 117 | 9 | -41 | -33 | 0.001 | 0.62 | 0.00001 | 0.00027 |  |  |
|  | L cerebral peduncle | 83 | -9 | -29 | -20 | 0.003 | 1.45 | -0.00012 | 0.00000 |  |  |
|  | R superior longitudinal fasciculus | 77 | 54 | -50 | -5 | 0.001 | 0.90 | 0.00033 | 0.00037 |  |  |
|  | R optic radiation | 62 | 16 | -70 | 17 | 0.001 | 1.02 | -0.00015 | -0.00007 |  | -0.871* |
|  | L anterior thalamic radiation | 59 | -3 | -22 | -13 | 0.002 | 1.76 | -0.00001 | 0.00006 |  |  |
|  | R forceps major | 51 | 20 | -82 | 26 | 0.001 | 1.32 | 0.00016 | 0.00022 |  | 0.455* |
|  | R inferior longitudinal fasciculus | 37 | 37 | -75 | 17 | 0.004 | 1.19 | 0.00023 | 0.00028 |  |  |
|  | Callosal body | 37 | 8 | 22 | -12 | 0.003 | 0.80 | 0.00006 | 0.00022 |  |  |
|  | R inferior longitudinal fasciculus | 36 | 37 | -78 | 26 | 0.001 | 1.20 | -0.00003 | 0.00007 |  |  |
|  | L corticospinal tract | 36 | -6 | -26 | -30 | 0.002 | 0.73 | -0.00031 | -0.00026 |  |  |
|  | R inferior fronto-occipital fasciculus | 33 | 22 | -81 | 33 | 0.004 | 1.60 | -0.00011 | -0.00003 |  |  |
|  | Unclassified | 30 | 12 | -43 | -17 | 0.004 | 0.92 | -0.00021 | -0.00009 |  |  |
|  | Unclassified | 25 | 15 | -50 | 47 | 0.001 | 1.33 | 0.00024 | 0.00030 | -0.651* |  |
|  | R inferior longitudinal fasciculus | 25 | 39 | -43 | -7 | 0.003 | 1.07 | 0.00004 | 0.00011 | -0.422* |  |
|  | Unclassified | 22 | 40 | -55 | -40 | 0.0004 | 0.66 | -0.00018 | -0.00009 |  |  |
|  | R superior longitudinal fasciculus | 21 | 55 | 6 | 18 | 0.002 | 1.47 | -0.00018 | -0.00008 |  |  |
|  | R superior longitudinal fasciculus | 19 | 48 | -33 | 42 | 0.002 | 1.28 | -0.00019 | -0.00009 |  |  |
|  | Unclassified | 19 | 15 | -40 | -22 | 0.003 | 1.46 | -0.00052 | -0.00028 |  |  |
|  | R corticospinal tract | 18 | 14 | -57 | -28 | 0.004 | 0.98 | 0.00017 | 0.00024 | -1.472** |  |
|  | Unclassified | 14 | 11 | -78 | 24 | 0.002 | 1.04 | -0.00018 | -0.00008 |  |  |
|  | R anterior thalamic radiation | 14 | 12 | -16 | -4 | 0.002 | 1.09 | 0.00021 | 0.00024 |  |  |
|  | Unclassified | 13 | 55 | -33 | 13 | 0.002 | 0.63 | -0.00002 | 0.00003 |  |  |
|  | R anterior thalamic radiation | 12 | 9 | -18 | 2 | 0.004 | 1.22 | -0.00014 | -0.00008 | 0.539** |  |
|  | Unclassified | 12 | 16 | -69 | -31 | 0.002 | 1.54 | -0.00045 | -0.00042 |  |  |
|  | R superior longitudinal fasciculus | 11 | 46 | -10 | 27 | 0.002 | 1.45 | 0.00000 | 0.00002 |  |  |
|  | R uncinate fasciculus | 10 | 35 | 1 | -17 | 0.003 | 1.00 | 0.00005 | 0.00011 |  |  |
|  | Unclassified | 10 | 54 | -26 | 39 | 0.002 | 0.79 | -0.00036 | -0.00028 |  |  |
|  |  |  |  |  |  |  |  |  |  |  |  |
| *Note.* WM FA = White matter fractional anisotropy, WM MD = White matter mean diffusivity. BM = *Bacopa monnieri* group. Some regions labelled as unclassified as JHU ICBM-DTI-81 Labels and JHU White-Matter Tractography Atlas do not include all white matter tracts. Local maximum coordinates in MNI space.  ^a^ age added as a covariate  ^b^ age and years of education added as a covariate  ^±^ standardised betas for clusters that significantly predicted spatial working memory original stimulus reaction time  * *p*<.05, ** *p*<.001 | | | | | | | | | | | |

| **Supplementary Table 2**. Uncorrected effects (*p* < .005) for measures of gray matter microstructure | | | | | | | | | | | |
| --- | --- | --- | --- | --- | --- | --- | --- | --- | --- | --- | --- |
|  |  |  | Local maximum | | |  |  | Mean change in clusters | | Step-wise regression *β*^±^ | |
| Measure | Region | Voxels | x | y | z | Max 1-p | T-value | Placebo | BM | Placebo | BM |
| **GM ND**^a^ |  |  |  |  |  |  |  |  |  |  |  |
| Placebo > BM | R temporal pole | 68 | 33 | 7 | -29 | 0.001 | 2.50 | 0.02600 | -0.05626 |  |  |
|  | L lingual gyrus | 18 | -18 | -48 | -11 | 0.001 | 6.29 | 0.03925 | -0.05947 |  |  |
|  | R planum polare | 17 | 43 | -8 | -14 | 0.001 | 2.02 | 0.02185 | -0.03161 | -0.595* |  |
|  | L lingual gyrus | 11 | -19 | -49 | -4 | 0.002 | 1.01 | 0.04248 | -0.01403 |  |  |
|  |  |  |  |  |  |  |  |  |  |  |  |
| Placebo < BM | L precuneous cortex | 20 | -4 | -48 | 52 | 0.002 | 2.63 | -0.03351 | 0.04645 |  |  |
|  |  |  |  |  |  |  |  |  |  |  |  |
| **GM ODI** |  |  |  |  |  |  |  |  |  |  |  |
| Placebo > BM | L caudate | 29 | -17 | 23 | -4 | 0.001 | 2.60 | 0.03528 | -0.06089 |  |  |
|  | L putamen | 11 | -26 | 2 | 1 | 0.004 | 1.51 | 0.03908 | -0.11351 | 0.579* |  |
|  |  |  |  |  |  |  |  |  |  |  |  |
| Placebo < BM | L posterior cingulate gyrus | 34 | -2 | -19 | 36 | 0.003 | 1.96 | -0.04300 | 0.03705 |  |  |
|  | L central opercular cortex | 22 | -46 | -9 | 10 | 0.002 | 2.36 | -0.03423 | 0.05380 |  |  |
|  | R central opercular cortex | 21 | 51 | -19 | 10 | 0.0002 | 3.46 | -0.05695 | 0.05145 |  |  |
|  | L anterior cingulate gyrus | 18 | -4 | -3 | 43 | 0.001 | 2.80 | -0.03364 | 0.05996 |  |  |
|  | R central opercular cortex | 15 | 47 | -5 | 7 | 0.002 | 3.19 | -0.05018 | 0.04687 |  |  |
|  | L anterior cingulate gyrus | 13 | -2 | 0 | 38 | 0.002 | 2.37 | -0.05060 | 0.02791 |  |  |
|  | R central opercular cortex | 12 | 56 | -4 | 11 | 0.001 | 2.43 | -0.03975 | 0.05138 |  |  |
|  | L frontal orbital cortex | 12 | -22 | 29 | -21 | 0.001 | 2.40 | -0.03868 | 0.05361 |  | -0.747* |
|  | L temporal pole | 11 | -41 | 7 | -26 | 0.0002 | 3.65 | -0.06720 | 0.05159 |  |  |
|  | R central opercular cortex | 10 | 37 | 5 | 12 | 0.001 | 3.13 | -0.07077 | 0.03947 |  |  |
| *Note.* GM ND = Gray matter neurite density, GM ODI = Gray matter orientation dispersion index, BM = *Bacopa monnieri* group. Local maximum coordinates in MNI space.  ^a^ age added as a covariate  ^±^ standardised betas for clusters that significantly predicted spatial working memory original stimulus reaction time  * *p*<.05, ** *p*<.001 | | | | | | | | | | | |
